# Toxicity and Its Mechanism Study of *Arecae semen* Aqueous Extract in Wistar Rats by UPLC-HDMS-Based Serum Metabolomics

**DOI:** 10.1155/2020/2716325

**Published:** 2020-01-29

**Authors:** Zhe Jia, Ting Han, Qinghua Lin, Wenjia Qu, Tianying Jia, Mengnan Liu, Haili Wang, Jieping Xin, Xinfang Xu, Xiangri Li

**Affiliations:** ^1^School of Chinese Materia Medica, Beijing University of Chinese Medicine, Higher Education Park, Fangshan District, Beijing 102488, China; ^2^Beijing Key Laboratory for Quality Evaluation of Chinese Materia Medica, Beijing University of Chinese Medicine, Beijing, China; ^3^College of Pharmacy, Guangxi University of Chinese Medicine, Guangxi Province, Nanning, China

## Abstract

**Background:**

*Arecae semen* (AS) is officially recorded in Chinese Pharmacopoeia and it is known for its multiple functions, including antidepressive, antioxidant, anti-inflammatory, and cholesterol-lowering effects, which have been confirmed by modern pharmacological study. Previous study in our laboratory showed that long-term oral administration of *Arecae semen* (AS) is officially recorded in Chinese Pharmacopoeia and it is known for its multiple functions, including antidepressive, antioxidant, anti-inflammatory, and cholesterol-lowering effects, which have been confirmed by modern pharmacological study. Previous study in our laboratory showed that long-term oral administration of *Hypothesis*. The aim of this work was to characterize the metabolome, evaluate the metabolic changes, and study the mechanisms of the toxicity induced by different treatment doses of ASAE via metabolomics.

**Methods:**

Wistar rats were administered orally two different doses of ASAE (1500 and 4500 mg/kg/d) for 30 days. The investigation was carried out to evaluate the safety of ASAE. And, the UPLC-HDMS-based serum metabolomics in conjunction with multivariate statistical techniques was applied to investigate the serum metabolite profile and potential markers of toxicity induced by different doses of ASAE.

**Results:**

Coupled with blood biochemistry and histopathology results, the significant difference in metabolic profiling was observed between 1500 and 4500 mg/kg/d dosages of ASAE-treated rats and normal rats by using pattern recognition analysis, indicating that changes in serum metabolites must have occurred. Some significant changed metabolites such as arachidonic acid, linoleic acid, stearic acid, and LPC (18 : 1) have been found and identified. These biochemical changes in serum metabolites are related to the perturbation of linoleic acid metabolism, arachidonic acid metabolism, glycerophospholipid metabolism, and purine metabolism, which may be helpful to further understand the cardiotoxicity and neurotoxicity of ASAE.

**Conclusion:**

The study shows that the metabolomic method may be a valuable tool for studying the essence of toxicity induced by traditional Chinese medicine.

## 1. Introduction

Metabolomics is commonly applied to discover potential biomarkers and biological mechanistic information by charactering the metabolic profiling of low-molecular-weight metabolites in biosamples [1]. It offers an alternative method for monitoring the biochemical changes associated with the generation and development of the influences resulting from endogenous and exogenous factors [2]. The change of all metabolites rapidly determined in biological samples, such as serum, plasma, and urine, can reflect the physiological status of the organism systematically. Thus, metabolomics approach has been enormously used in toxicology research [3, 4], biomedical, as well as nutritional studies.

Toxicity is a major concern in the treatment of TCM. The constituents of TCM are highly complex. And TCM has the feature of integrity and systemic, which is well coincident with the systemic thinking and strategy of the metabolomics [5]. Metabolomics can be used to analyze the differences of the blood and urine metabolites of different TCM syndromes, which can make the diagnosis of TCM syndromes more scientific and quantitative, and the uncertainty of the subjective factors can be avoided. As a convenient and effective tool, metabolomics methods have been used in the evaluation of the toxicology of TCM [2, 6, 7].

It is really a great challenge for identifying the metabolites in complex mixtures. Advanced analytical technologies play a significant role in comprehensive endogenous metabolite profiling. Due to the high sensitivity and selectivity, UPLC-HDMS has become a rapid and reliable tool for metabolomics studies, which has been widely used in different laboratories around the world [8].

AS, the seed of *Areca catechu* Linn. (Family: Palmaceae) Palm, has been widely used for medicinal purposes for more than 2000 years in South Asian countries [9]. AS is one of the clinically used common drugs for the treatment of gastrointestinal, edematous, and parasitic diseases [10]. Modern pharmacological studies demonstrated that AS has good antidepressive, antioxidant, anti-inflammatory, antiaging, analgesic, and plasma cholesterol-lowering effects [11–15]. Recent researches indicated that arecoline, the major alkaloid in AS, is an agonist for the muscarinic receptor, and has a stimulating effect on parasympathetic and cardiovascular system [16], which is the major source of toxicity at the same time [17].

The common adverse reactions of preparations of AS included nausea and vomiting, abdominal pain, and dizziness [18]. The LD_50_ value of ASAE in mice was 3.67 g/kg (p.o.) [17]. Furthermore, the ASAE (200 *μ*g/mL) and arecoline (10 *μ*g/mL) could significantly upregulate the mRNA expression of the *c-jun* proto-oncogene in oral mucosal fibroblasts, indicating that persistent induction of *c-jun* proto-oncogene by the areca nut and arecoline may be one of the mechanisms for oral squamous cell carcinoma [19, 20]. In addition, it has been reported that the ASAE (40–80 *μ*g/mL) causes a significant proliferative effect on oral mucosal fibroblasts (OMF) via upregulation of the expression of ICAM-1 [21, 22]. ASAE (5 and 20 mg/kg) and arecoline (5 and 20 mg/kg, p.o.) also increase the serum levels of ALT, AST, and ALP in mice [23, 24]. Besides, it has been reported that ASAE resulted in signs of reproductive toxicity including decreases in sperm counts and sperm motility and the induction of substantial abnormalities in sperm morphology [25–27]. Our previous study showed that ASAE at the dose of 750 mg/kg/day was safe, while long-term (30 days) oral administration of ASAE at higher dosage levels (1500 mg/kg and 4500 mg/kg) showed dose-dependent toxic effects [28]. Besides, we focused on the toxicity induced by different duration of administration (7-day, 14-day, and 30-day) of ASAE at a dose of 4500 mg/kg/d. It indicated that *Arecae* semen possessed time-dependent cardiotoxicity and the metabolomics approach is a useful tool to study the toxicity in TCM [29].

In this study, UPLC-HDMS-based serum metabolomics combined with multivariate statistical techniques was used to characterize the serum metabolite profile and evaluate the metabolic changes and potential markers of toxicity induced by different doses of of ASAE (1500 and 4500 mg/kg/d). The results may provide valuable and in-depth information about the toxic effects of AS on Wistar rats, which will be beneficial for future safe application of this herb in clinic.

## 2. Materials and Methods

### 2.1. Plant Material and Reagents

AS was purchased from Beijing Tongrentang, Bozhou. Slices of Chinese Crude Drugs Co., Ltd. were prepared and identified as the ripe seed of *Areca catechu* L. by Professor Xiangri Li (School of Chinese Materia Medica, Beijing University of Chinese Medicine). The samples were deposited in the specimen cabinet of traditional Chinese medicine at Beijing University of Chinese Medicine. LC-grade acetonitrile was purchased from the Baker Company (Mallinckrodt Baker Inc., Phillipsburg, NJ, USA). Ultrahigh purity water was prepared using a Milli-Q water purification system (Millipore Corp., Billerica, MA, USA). We followed the methods of author links open overlay panel Lin et al. [29].

### 2.2. AS Aqueous Extract Preparation

25 kg AS smashed into coarse particle using hammer mill, was extracted with 150 L distilled water for 1 h, and ﬁltered. Then, the supernatant was collected for further processing and the residue was extracted with the same process. The aqueous extract was combined and evaporated under vacuum to yield the dark brown extract (1 g of the extract corresponding to 4.91 g of AS). Then, the ASAE was stored in an amber bottle and kept at −80°C until further use. The ASAE was thawed and freshly prepared with distilled water based on the daily requirement before oral administration. And the quantification of chemical compounds by HPLC analysis was shown in Supplementary Materials.

### 2.3. Animals and Treatment

Adult male Wistar rats were purchased from SPF (Beijing) Biotechnology Co., Ltd., Beijing, China. The animals were provided with commercial food and water *ad libitum* at the standard laboratory condition (temperature of 25°C ± 2°C, relative humidity of 55 ± 5% and 12 h light-dark cycle). After 5 days of acclimatization, the rats (180–200 g) were divided into one control group and two treated groups (*n* = 6 in each group). During the 30-day treatment period, the control group was orally administrated with distilled water, and the treated groups were administrated with ASAE at 1500 and 4500 mg/kg/day based on body weight. According to the Chinese Pharmacopoeia, the maximal dose of AS is 60 g/day for an adult (70 kg). In view of the results of preliminary experiment and the purpose of exhaustively exploring the toxicity of AS, the dosages of ASAE were set at 1500 and 4500 mg/kg/day, which were 9 and 27 times of the clinical dosage, respectively. The rats were weighed every 3 days. Mortality and clinical reaction of all the rats from different groups were observed before and after administration including death, piloerection, breathing, behavior, excretion, secretion, and other possible changes.

The experiment was in accordance with the Animal Management Rules of the Ministry of Science and Technology of the People's Republic of China for experimental care and use of animals and approved by the Animal Ethics Committee of Beijing University of Traditional Chinese Medicine.

### 2.4. Sample Collection and Preparation

After 30 days of treatment, the rats were fasted overnight but allowed to access water freely before the autopsy. Finally, the blood samples were obtained from abdominal aorta, and the different tissues were rapidly removed for pathological staining and assay under anesthesia (pentobarbital, 1 mL/kg body weight, *i.p.*). The serum obtained from blood samples by centrifugation (3000 rpm, 10 min, 4°C) was stored in the refrigerator at −80°C. The serum sample from each rat was divided into two parts, one for UPLC-HDMS analysis and the other for biochemical analysis.

### 2.5. Serum Biochemistry

The serum samples for biochemistry test were thawed at room temperature for biochemical examination. ALT and AST activities were measured using the corresponding detection kits (Nanjing Jiancheng Bioengineering Institute, Nanjing, China), which were used to determine the effect of ASAE on liver. BUN and Cr activities were inspected by using kits (Beijing Solarbio Science & Technology Co., Ltd., Beijing, China), which could reflect the effect of ASAE on nervous system. CK detection kit (Nanjing Jiancheng Bioengineering Institute, Nanjing, China) was used to evaluate the effect of ASAE on heart.

### 2.6. Histopathology

After dissection to remove the fat and connective tissue, the following organs were obtained: liver, kidney (the right), spleen, heart, and lungs. All the organs were visually inspected and then fixed in 10% neutral buffered formalin at room temperature, gradually dehydrated in ethanol, and embedded in paraffin. Paraffin blocks were cut into 4 *μ*m sections, and the sections were stained with hematoxylin and eosin (H&E). The sections were examined under an Olympus PROVIS AX70 light microscope (Tokyo, Japan) to detect any morphological changes in the tissue using a Nikon camera.

### 2.7. Chromatographic Separation and Mass Spectrometry

Prior to analysis, the serum samples were thawed at room temperature. Acetonitrile (600 *μ*L) was added into serum (200 *μ*L) and vortex-mixed for 3 min for protein precipitation. After centrifugation at 14,000 rpm for 10 min at 4°C, the supernatant (600 *μ*L) was pipetted out and dried with nitrogen. The residues were dissolved in 100 *μ*L acetonitrile: water (80 : 20). Then, the samples were vortex-mixed for 3 min and the supernatant was collected for UPLC-HDMS analysis after centrifugation (14000 rpm, 10 min, 4°C).

Chromatographic separation was performed on a C_18_ column (2.1 × 100 mm, 1.6 *μ*m, Waters, Ireland) with an Accela 600 pump LC system (Thermo Scientific, Bremen, Germany) equipped with a binary pump and an autosampler. The UPLC mobile phase consisted of acetonitrile with 0.1% formic acid (solution B) and water with 0.1% formic acid (solution A). The flow rate was 0.30 mL/min and the linear gradient elution program was as follows: 0–0.5 min, 1% B; 0.5–20.0 min, 1–95% B; 20.0–21.0 min, 95% B; 21.0–24.0 min, 95–1% B. The analytical column and autosampler were maintained at 40°C and 4°C. 1 *μ*L aliquot of each sample was injected for analysis.

Mass spectrometry was performed on an LTQ-Orbitrap mass spectrometer (Thermo Scientific, Bremen, Germany) connected to the UPLC instrument via ESI interface. Samples were analyzed in both negative mode and positive mode with the tune method set as follows: sheath gas (nitrogen) flow rate of 30 arb, aux gas (nitrogen) flow rate of 5 arb, spray voltage of 4.0 kV, capillary temperature of 350°C, capillary voltage of 25 V, and tube lens voltage of 110 V. The measured masses were within 5 ppm of the theoretical masses. The scan range was from 50 to 1200 *m*/*z*.

### 2.8. QC Samples and Method Validation

An equal volume of serum from all the samples was pooled to get a pooled QC sample, and then the QC sample was processed by the same method as the sample preparation described above.

The precision of instrument, method repeatability, and system stability were validated by QC samples to make sure that the method of UPLC-HDMS could meet the requirement of metabolomics. The 5 replicated injections from the same QC sample and 5 injections from the different QC samples were used to evaluate the precision of instrument and the method repeatability, respectively. The system stability was verified by injecting a QC sample every 10 samples during the analysis.

### 2.9. Data Analysis and Statistical Analysis

The raw UPLC-MS data were imported into XCMS online software and then processed with default settings to complete baseline correction, peak discrimination and alignment, and retention time correction. The ion intensities or peak area for each peak detected was also normalized within each sample to the sum of the peak intensities in that sample. The unsupervised PCA and the supervised OPLS-DA were performed on SIMCA-P 13.0 (Umetrics, Umea, Sweden). The differential metabolites were selected on the basic of two criteria: (a) the value of VIP from the OPLS-DA model should be satisfied with VIP > 1 and (b) the result from the Student's *t*-test should meet the criteria of *P* < 0.05 by using SPSS 17.0 (IBM, New York, USA).

According to the previous reports [30–33], the identification of potential biomarkers were based on the MS/MS fragment ion and matched with the structure message from HMDB (http://www.hmdb.ca/), METLIN (http://metlin.scripps.edu/), and ChemSpider (http://www.chemspider.com/). The pathway analysis of potential biomarkers was performed by MetaboAnalyst (http://www.metaboanalyst.ca/) and KEGG (http://www.genome.jp/kegg/) [34].

## 3. Results and Discussion

### 3.1. HPLC Analysis

The HPLC chromatogram of 1500 mg/kg/d ASAE sample was included in supplementary materials ([Supplementary-material supplementary-material-1]). The concentrations of the alkaloids in the ASAE of the 1500 mg/kg/d and 4500 mg/kg/d groups are shown in Table 1.

We used HPLC analysis to determine the contents of four alkaloids, which are the main active ingredients in AS. By doing this, we could determine whether some reactions after administration are caused by a high content of a specific compound. It also provides a preliminary basis for further studies on the four alkaloids.

### 3.2. General Observations

Compared with the control group, the rats in the treated groups exhibited diarrhea, less physical activity, tremors, and body curl up, which suggested that ASAE have effects on the nervous system.

**Figure 1:**
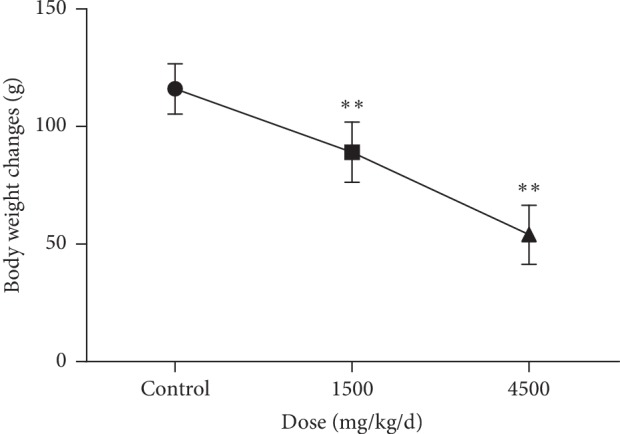
Effects of ASAE on the body weight changes of rats during the treatment period study (*n* = 6). Values are mean ± SD. ^*∗∗*^*P* < 0.01 significant difference compared with the control group (One-way ANOVA followed by Dunnett's test).

As shown in Figure 1, compared with drug-treated (1500 mg/kg and 4500 mg/kg) and control groups, a significant dose-dependent decrease was observed in the body weights of male rats, which indicated that ASAE had inhibition effects on the normal growth of rats.

### 3.3. Biochemical Results and Histopathology

**Figure 2:**
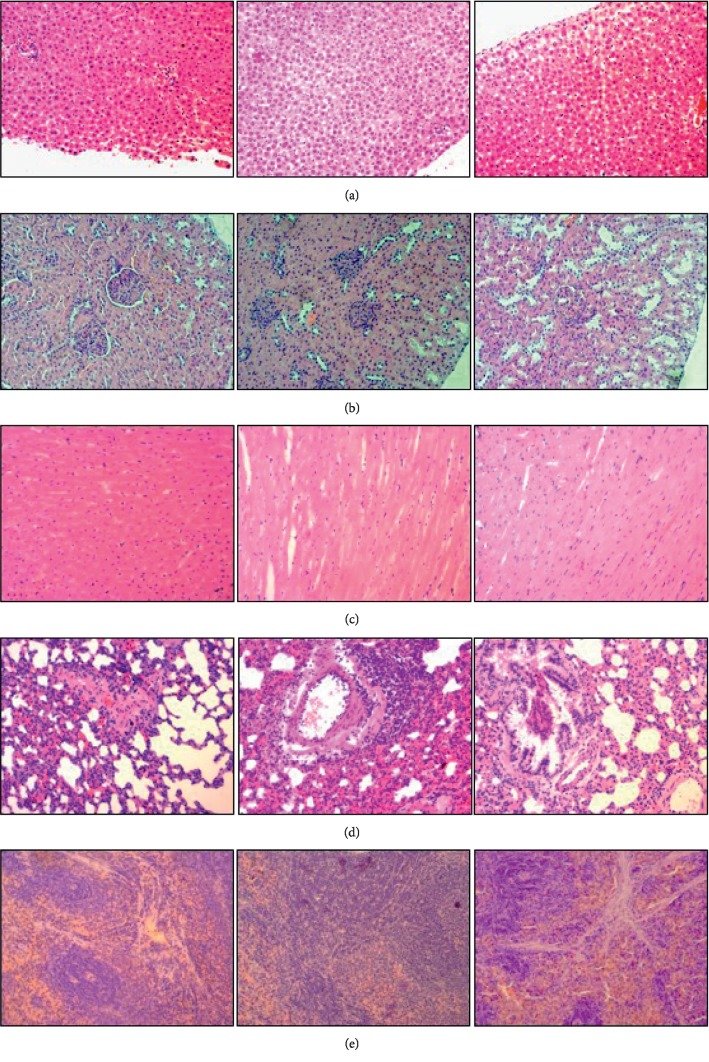
Histopathological tissue sections of Wistar rats. (a) Liver: 0, 1500 mg/kg/d, 4500 mg/kg/d. (b) Kidney: 0, 1500 mg/kg/d, 4500 mg/kg/d. (c) Heart: 0, 1500 mg/kg/d, 4500 mg/kg/d. (d) Lung: 0, 1500 mg/kg/d, 4500 mg/kg/d. (e) Spleen: 0, 1500 mg/kg/d, 4500 mg/kg/d.

As shown in Table 2, there were no significant differences (*P* > 0.05) in the levels of AST, BUN, and Cr between the treated and control groups. The level of ALT in 4500 mg/kg group (48.50 U/L) was significantly decreased (*P* < 0.05) compared with the control group (67.90 U/L), which suggested that AS was beneficial for liver to some extent. Moreover, the histopathological changes of liver, kidney, lung, and spleen were not significant in the treated and control groups (Figures 2(a), 2(b), 2(d) and 2(e)), which were in accord with the biochemical results and our previous study [28]. However, other researchers had come up with AS extracts that were toxic to hepatocytes in mice [35], which were inconsistent with our study. Therefore, further researches are still needed to provide more in-depth insight into the hepatotoxicity of AS.

The level of CK in 4500 mg/kg group (1695.20 U/L) was significantly increased (*P* < 0.05) compared with the control group (996.75 U/L), which suggested that AS might possess cardiotoxicity to some extent (Table 2). However, no significant abnormal structures in heart sections were observed in the treated groups (Figure 2(c)), which may be due to the morphology changes being less sensitive than the biochemical alterations.

### 3.4. Validation of the UPLC-HDMS Method

The RSD of *t*_R_ and peak intensities for the selected ions from QC samples were calculated to evaluate the method performance, and the detailed information of the selected ions is shown in [Supplementary-material supplementary-material-1].

As shown in [Supplementary-material supplementary-material-1], the RSD of the retention times for precision, stability, and repeatability were 0.05–0.21%, 0.11–1.72%, and 0.02–0.67% in negative ion mode and 0.03–0.59%, 0.02–0.82%, and 0.04–0.56%, respectively, in positive ion mode; the RSD of the peak intensities for precision, stability, and repeatability were 4.72–7.19%, 5.23–8.39%, and 4.92–9.31% in negative ion mode and 2.23–8.38%, 2.11–7.45%, and 2.62–7.21%, respectively, in positive ion mode. 20% is acceptable RSD for accuracy and precision in metabolomics [36]. Therefore, the results described above demonstrated that the proposed method was accurate and robust for further studies.

### 3.5. Multivariate Statistical Analysis

**Figure 3:**
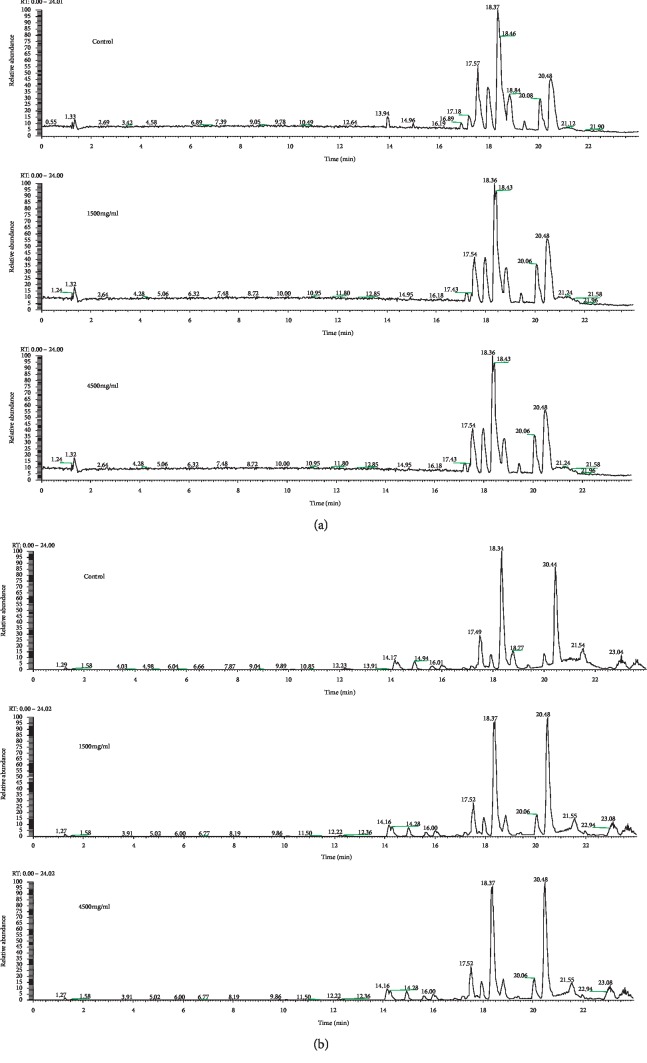
Representative base peak intensity obtained in the ESI negative ion mode (a) and positive ion mode (b) of rat serum after oral administration of ASAE.

The representative BPI chromatograms of the control and treated groups in both negative and positive modes (Figures 3(a) and 3(b)) were obtained from UPLC-HDMS analysis, respectively. Although some of the differences could be observed directly from the BPI chromatograms, the subtle metabolism variations still required to be further investigated by multivariate statistical analysis, such as PCA and OPLS-DA.

**Figure 4:**
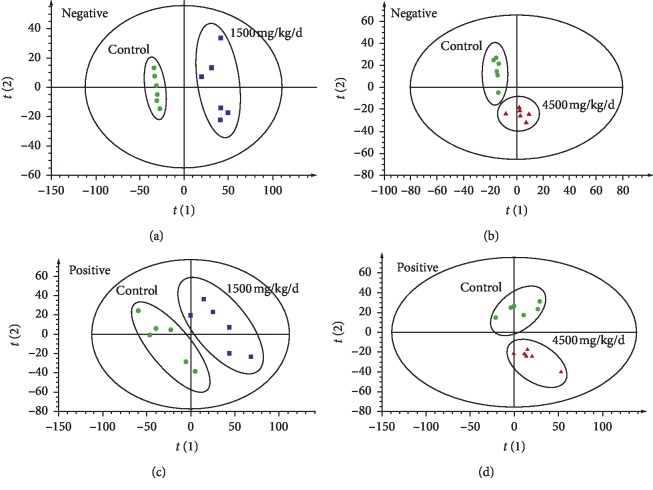
PCA plots with the scores of the first two principal components (a) exhibiting the scatter between 1500 mg/kg/d dose of ASAE-induced group (blue square) and the control group (green circle) in the ESI negative ion mode, *R*^2^*X* = 0.640, *Q*^2^(cum) = 0.484, (b) exhibiting the scatter between 4500 mg/kg/d dose of ASAE-induced group (red triangle) and the control group (green circle) in the ESI negative ion mode, *R*^2^*X* = 0.659, *Q*^2^(cum) = 0.170, (c) exhibiting the scatter between 1500 mg/kg/d dose of ASAE-induced group (blue square) and the control group (green circle) in the ESI positive ion mode, *R*^2^*X* = 0.487, *Q*^2^(cum) = 0.135, and (d) exhibiting the scatter between 4500 mg/kg/d dose of ASAE-induced group (red triangle) and the control group (green circle) in the ESI positive ion mode, *R*^2^*X* = 0.548, *Q*^2^(cum) = 0.0519.

The PCA, as an unsupervised analysis, is usually performed to visualize clustering, trends or outliers among the observations at the first step of metabolomics analysis. In Figure 4, the PCA score plot showed that the separation between control and two different doses of ASAE-treated groups was obvious in positive mode and negative mode, respectively. The results indicated that there were different metabolic profilings between the control and two different doses of ASAE-treated groups.

**Figure 5:**
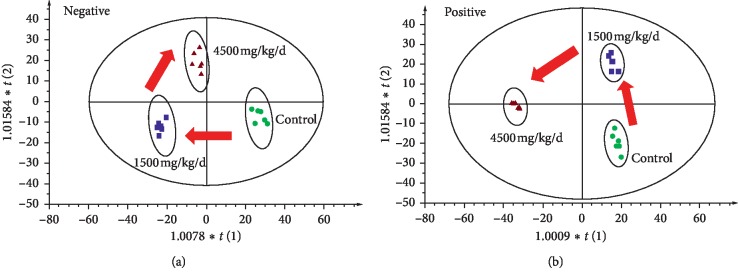
OPLS-DA score plots derived from the UPLC-MS data from serum samples after different doses of ASAE treatment: (a) exhibiting the scatter between 1500 mg/kg/d (blue square) and 4500 mg/kg/d (red triangle) doses of ASAE-induced groups and the control group (green circle) in the ESI negative ion mode, *R*^2^*X* = 0.734, *R*^2^*Y* = 0.989 and *Q*^2^(cum) = 0.887 and (b) exhibiting the scatter between 1500 mg/kg/d (blue square) and 4500 mg/kg/d (red triangle) doses of ASAE-induced groups and the control group (green circle) in the ESI positive ion mode, *R*^2^*X* = 0.629, *R*^2^*Y* = 0.994 and *Q*^2^(cum) = 0.900.

**Figure 6:**
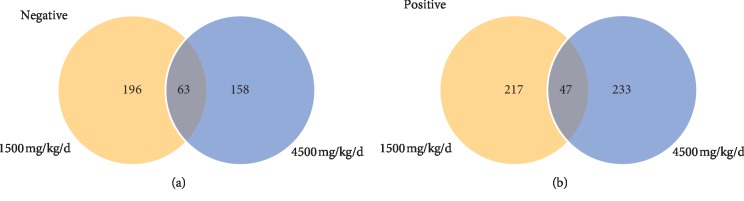
Venn diagrams of biomarkers from serum samples of different doses of ASAE treatment groups: (a) The ESI negative ion mode and (b) the ESI positive ion mode.

Unlike the PCA approach, the OPLS-DA is a supervised method and frequently employed to increase the separation and search for the potential biomarkers by the contribution of the variables in group separation. As shown in Figure 5, the score plots of OPLS-DA showed that there were clear group discriminations and dose-dependent dynamic changes that gradually moved far away from the control group position, no matter in negative mode or positive mode. The model statistics indicated no signs of overfitting, and the model was valid. The results suggested that compared with the control, the variance of metabolites in treated rats was enlarged over different doses of ASAE. Compared with the control group, 196 and 158 differential metabolisms (VIP > 1 and *P* < 0.05) were screened from the 1500 mg/kg/d and 4500 mg/kg/d doses of ASAE-induced groups in the ESI negative ion mode, respectively. Besides, 217 and 233 biomarkers were screened from the low and high dose of ASAE-induced groups in the ESI positive ion mode, respectively. Considering the stability of the biomarkers at different doses of ASAE in the treated groups, Venn diagram was used to separately integrate the metabolites information. At last, 63 and 47 metabolites were selected as the candidates in the negative mode and positive mode, respectively ([Supplementary-material supplementary-material-1] and Figure 6).

### 3.6. Identification of Biomarkers

Metabolite peaks were assigned by MS^E^ analysis or interpreted with available biochemical databases, such as METLIN (https://metlin.scripps.edu/), HMDB (http://www.hmdb.ca/), ChemSpider (http://www.chemspider.com/), and MassBank (https://massbank.eu/MassBank/). In the Supplementary Materials, a potential biomarker with an *m*/*z* of 255.2332 is used as an example to illustrate the identification process ([Supplementary-material supplementary-material-1]). By using the same method described, 16 metabolites (12 from the negative mode and 4 from the positive mode) were identified and tentatively regarded as the potential biomarkers in the treated groups induced by ASAE (Table 3).

### 3.7. Biochemical Interpretation

**Figure 7:**
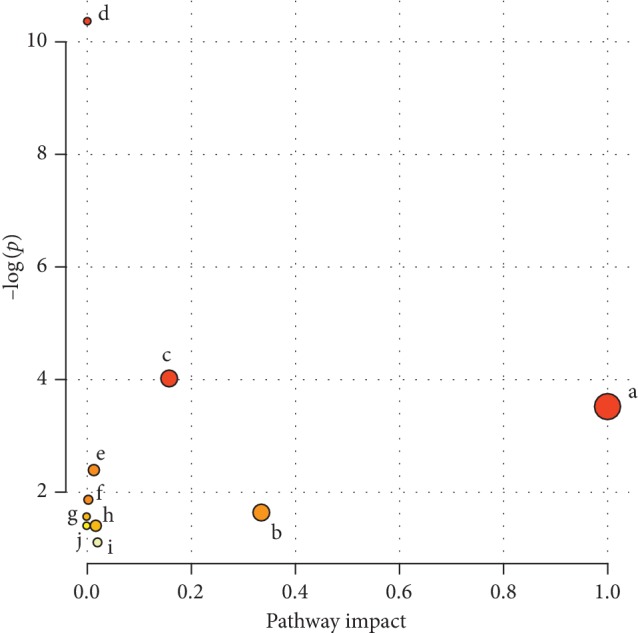
Metabolic pathway analysis based on potential markers identified in serum metabonomics. a, linoleic acid metabolism; b, arachidonic acid metabolism; c, glycerophospholipid metabolism; d, biosynthesis of unsaturated fatty acids; e, fatty acid biosynthesis; f, purine metabolism; g, glycerolipid metabolism; h, fatty acid elongation in mitochondria; i, fatty acid metabolism; j, primary bile acid biosynthesis.

MetPA is a part of many functions in MetaboAnalyst network database. It can visualize the metabolic pathway information of potential biomarkers with the help of METLIN, HMDB, and KEGG database. In this study, sixteen potential biomarkers were identified in the ASAE-treated groups using the UPLC-HDMS based on serum metabolomics. The 16 identified potential biomarkers in this experiment were analyzed by MetPA, and the results are shown in Figure 7 and Table 4. And the related pathway of those biomarkers included linoleic acid metabolism, arachidonic acid (AA) metabolism, glycerophospholipid metabolism, and purine metabolism.

## 4. Discussion

In our study, the techniques of metabolomics were combined with phenotypic responses and biochemical assays to confirm the target organ and understand the toxic effects of AS in rats. The interpretation of the metabolism contributes to further understand the toxicity of ASAE in different doses and explore its underlying mechanism.

According to the result of HPLC analysis, the content of guvacine was a lot higher than that of three other alkaloids. Based on the LC_50_ of four alkaloids on zebrafish in our laboratory, the toxicity of guvacine is inferior to arecoline, which is the most toxic. There are few reports about the toxicity of guvacine. But for arecoline, it has been widely studied due to its carcinogenic potential [20, 37–41]. Besides, it has parasympathetic effect, including euphoria, central nervous system stimulation, vertigo, salivation, miosis, tremor, and muscarinic effects [42]. Therefore, we deduced that the cardiotoxicity of AS described in this study might be largely attributable to arecoline.

Linoleic acid, an important polyunsaturated fatty acid in brain tissue is related to symptoms of growth retardation [43], cardiac toxicity [44], and neurotoxicity. In this experiment, compared with the control group, the body weight changes of rats in treated groups showed significant dose-dependent decreases. During the 30 days of oral administration of ASAE, food and water were supplied sufficiently and all the rats were given free diet. Therefore, we could infer that it is the AS inducing the toxicity of growth retardation in the treated groups, which may cause the difference of endogenous metabolites. In this work, the AS-treated rats exhibited tremors and bodies curled up in a dose-dependent manner. These neurotoxicity symptoms may be attributed to the reduction of linoleic acid. Moreover, the level of CK increased significantly in the AS-treated groups, suggesting that the AS exhibited cardiotoxicity to some extent.

Arachidonic acid, a derivative of linoleic acid, is an essential polyunsaturated fatty acid, which cannot be synthesized in the body, but must be obtained from the daily diet [45]. As a constituent exists mainly in the form of phospholipid in cell membrane, AA is the substrate for the synthesis of a range of biologically active compounds including prostaglandins, which can cause diarrhea by stimulating gastrointestinal smooth muscle. In our study, compared with the control group, the level of AA was significantly downregulated in a dose-dependent manner, which may be the major cause of diarrhea.

Glycerophospholipids (GPs), which are the critical components of the lipid bilayer of cellular membranes, play a key role in cell signaling and metabolism. Depending on the different substituents at the sn-3 position of the glycerol backbone, GPs fall into PC, PE, PG, PS, PI, PA, and cardiolipins [46, 47]. LPC is generated by hydrolysis or oxidation of PC [48]. Studies have demonstrated that acceleration of the lipid peroxidation of the cardiac systems may lead to heart arrhythmia [49, 50]. LPCs, as biomarkers of lipid peroxidation, were positively related to the level of lipid peroxidation [51, 52], which plays an important role in the pathogenesis of cardiac tissue [49]. In our study, compared with the control group, the level of LPCs was significantly upregulated in a dose-dependent manner. This result was consistent with the increased level of CK in the AS-treated groups (Table 2), which confirmed that the AS caused cardiotoxicity to some extent. And this result was in accordance with our previous study [29]. Nevertheless, AS, the seed of *Areca catechu* Linn, contains lipids as well. Objectively speaking, we could not rule out the possibility that the increased LPC in the blood may be from the absorbed constituents of AS.

Uric acid is the final product of purine metabolism. A growing body of evidence suggested that high levels of serum uric acid serve also as biomarkers for cardiovascular disease morbidity and mortality [53]. In our study, compared with the control group, the level of uric acid was upregulated in AS-treated rats (4500 mg/kg/d). This result also demonstrated that the AS causes cardiotoxicity to a certain degree. Moreover, it is reported that dysregulation of purine metabolism may be one of the causes of mental retardation or unexpected and often devastating neurological dysfunction [54], leading to tremors and body curl up.

## 5. Conclusions

In this study, by using biochemistry and histopathology methods, we found that long-term oral administration of ASAE caused cardiotoxicity and neurotoxicity in rats in a dose-dependent manner. Moreover, 16 potential biomarkers such as arachidonic acid, linoleic acid, stearic acid, LPC (18 : 1), and LPC (18 : 2) were identified in the serum of ASAE-treated rats by using metabolomics studies combined with multivariate data analysis. Those biomarkers are related to the perturbations of linoleic acid metabolism, arachidonic acid metabolism, glycerophospholipid metabolism, and purine metabolism. The detailed effect of AS on the mentioned metabolic pathways requires further research, and the insights gained are just preliminary in nature to elucidate the metabolic pathways that should receive enlarged interest in further studies. Genomics, transcriptomics and proteomics are needed to comprehensively study the underlying toxicological mechanisms of ASAE.

## Figures and Tables

**Table 1:** The concentrations of arecoline, guvacoline, arecaidine, and guvacine in the ASAE samples (*n* = 3).

Compounds	Contents (mg/mL)	
	1500 (mg/kg/d)	4500 (mg/kg/d)
Arecoline	0.67	2.35
Guvacoline	0.32	0.69
Arecaidine	0.41	1.12
Guvacine	1.22	4.23

**Table 2:** Effects of ASAE on biochemical parameters of rats in the study (*n* = 6).

	Control group	1500 (mg/kg)	4500 (mg/kg)
BUN (mmol/L)	4.25 ± 0.60	3.98 ± 1.17	4.93 ± 1.40
ALT (U/L)	67.90 ± 13.50	53.70 ± 15.11	48.50 ± 15.83^*∗*^
AST (U/L)	204.78.0 ± 17.36	197.56 ± 20.12	168.80 ± 19.24
CK (U/L)	996.75 ± 145.23	1275.60 ± 347.09	1695.20 ± 223.32^*∗*^
Cr (*μ*mol/L)	33.44 ± 4.16	35.50 ± 5.25	27.00 ± 4.34

Values are mean ± SD. ^*∗*^*P* < 0.05 significant difference compared with the control group (one-way ANOVA followed by Dunnett's test).

**Table 3:** The identification of potential biomarkers and their changes in rats induced by ASAE.

HMDB ID	Metabolites	Formula	*t*_R_ (min)	Exact mass (*m*/*z*)	Determined (*m*/*z*)	Calculated (*m*/*z*)	Error (ppm)	Ion from	Related pathways	Trends/(mg/kg/d)	
										1500	4500
HMDB0000220	Palmitic acid	C_16_H_32_O_2_	17.34	256.4241	255.2332	255.2324	3.1	[M − H]^−^	Fatty acid metabolism	↑*∗*	↑
HMDB0000673	Linoleic acid	C_18_H_32_O_2_	15.30	280.4455	279.2322	279.2324	−0.9	[M − H]^−^	Linoleic acid metabolism	↓*∗∗*	↓*∗∗∗*
HMDB0001043	Arachidonic acid	C_20_H_32_O_2_	16.28	304.4669	303.2324	303.2324	0	[M − H]^−^	Arachidonic acid metabolism	↓	↓*∗∗∗*
HMDB0003229	Palmitoleic acid	C_16_H_30_O_2_	14.43	254.4082	253.2168	253.2167	0.4	[M − H]^−^	Fatty acid metabolism	↓*∗∗∗*	↓
HMDB0000827	Stearic acid	C_18_H_36_O_2_	9.14	284.4772	283.2635	283.2637	−0.7	[M − H]^−^	Fatty acid metabolism	↓*∗∗*	↓*∗∗∗*
HMDB0010382	LysoPC (16 : 0)	C_24_H_50_NO_7_P	19.42	495.6301	494.3231	494.3252	−4.3	[M − H]^−^	Glycerophospholipid metabolism	↑	↑*∗*
HMDB0007853	LysoPA (16 : 0/0 : 0)	C_19_H_39_O_7_P	20.00	410.4825	409.2359	409.2361	−0.5	[M − H]^−^	Glycerolipid metabolism	↓*∗∗∗*	—
HMDB0000289	Uric acid	C_5_H_4_N_4_O_3_	1.35	168.1103	167.0205	167.0211	−3.6	[M − H]^−^	Purine metabolism	—	↑
HMDB0000619	Cholic acid	C_24_H_40_O_5_	13.62	408.5714	407.2801	407.2803	−0.5	[M − H]^−^	Primary bile acid biosynthesis	—	↑*∗*
HMDB0010381	LysoPC (15 : 0)	C_23_H_48_NO_7_P	18.38	481.6035	480.3084	480.3096	−2.5	[M − H]^−^	Glycerophospholipid metabolism	—	↑
HMDB0011482	LysoPE (0 : 0/20 : 1)	C_25_H_50_NO_7_P	18.82	507.6408	506.3224	506.3252	−5.5	[M − H]^−^	Glycerophospholipid metabolism	—	↑
HMDB0011481	LysoPE (0 : 0/20 : 0)	C_25_H_52_NO_7_P	20.54	509.6566	508.3387	508.3409	−4.3	[M − H]^−^	Glycerophospholipid metabolism	—	↑
HMDB0002815	LysoPC (18 : 1)	C_26_H_52_NO_7_P	12.68	521.6673	522.3534	522.3559	−4.8	[M + H]^+^	Glycerophospholipid metabolism	↑	↑*∗*
HMDB0010386	LysoPC (18 : 2)	C_26_H_50_NO_7_P	11.36	519.6515	520.3380	520.3403	−4.2	[M + H]^+^	Glycerophospholipid metabolism	↑	↑*∗*
HMDB0010397	LysoPC (20 : 5)	C_28_H_48_NO_7_P	11.33	541.6570	542.3215	542.3246	−5.7	[M + H]^+^	Glycerophospholipid metabolism	↑	↑*∗*
HMDB0010404	LysoPC (22 : 6)	C_30_H_50_NO_7_P	11.32	567.6943	568.3373	568.3403	−5.3	[M + H]^+^	Glycerophospholipid metabolism	↑	↑*∗*

↑: upregulation compared with control group, ↓: downregulation compared with control group. ^*∗*^*P* < 0.05, ^*∗∗*^*P* < 0.01, ^*∗∗∗*^*P* < 0.001.

**Table 4:** Pathway analysis of potential markers identified in serum metabonomics.

Pathway name	Total	Hits	*P*	−log(*p*)	FDR	Impact
Biosynthesis of unsaturated fatty acids	42	4	0.000079	9.4482	0.006385	0
Glycerophospholipid metabolism	30	2	0.014520	4.2322	0.58807	0.24197
Fatty acid biosynthesis	43	2	0.028857	3.5454	0.64258	0
Linoleic acid metabolism	5	1	0.031732	3.4504	0.64258	1
Purine metabolism	68	2	0.066940	2.7040	1	0.02838
Glycerolipid metabolism	18	1	0.110090	2.2065	1	0.01920
Fatty acid elongation in mitochondria	27	1	0.160980	1.8265	1	0
Arachidonic acid metabolism	36	1	0.209270	1.5641	1	0.32601
Fatty acid metabolism	39	1	0.224810	1.4925	1	0
Primary bile acid biosynthesis	46	1	0.260010	1.3470	1	0

Total: the number of all metabolites in the metabolic pathway. Hits: the number of differentiated metabolites selected in the metabolic pathway. Raw *P*: the original calculated *P* value of the enrichment analysis. FDR: the value of FDR in multiplex checking. Impact: the influence value calculated by path topology analysis.

## Data Availability

The data used to support the findings of this study are available from the corresponding author upon request.

## Supplementary Materials

Supplementary Materials Figure S1: the HPLC chromatograms of mixed standards (a) and 1500 mg/kg/d ASAE samples (b). (1) Arecoline; (2) guvacoline; (3) arecaidine; (4) guavacine. Figure S2: LTQ-Orbitrap mass spectra of the metabolite with MSE function at low energy and high energy for the biomarker at m/z 255.2332 in negative ion mode (a) and the product ion scan spectrum (MSE) for the biomarker in negative ion mode (b). Table S1: the analytical performance of the UPLC-HDMS method (*n* = 5). Table S2: the detailed information of selected ions from QC samples of the UPLC-HDMS method. Table S3: the detailed information of selected ions from QC samples of the UPLC-HDMS method.
