# Supplementary material for: Toxicity and Its Mechanism Study of Arecae semen Aqueous Extract in Wistar Rats by UPLC-HDMS-Based Serum Metabolomics
Source: Evid Based Complement Alternat Med. 2020 Jan 29;2020:2716325. doi: 10.1155/2020/2716325 (PMC7011391; doi:10.1155/2020/2716325)
Supplement: Supplementary Materials — Figure S1: the HPLC chromatograms of mixed standards (a) and 1500 mg/kg/d ASAE samples (b). (1) Arecoline; (2) guvacoline; (3) arecaidine; (4) guavacine. Figure S2: LTQ-Orbitrap mass spectra of the metabolite with MSE function at low energy and high energy for the biomarker at m/z 255.2332 in negative ion mode (a) and the product ion scan spectrum (MSE) for the biomarker in negative ion mode (b). Table S1: the analytical performance of the UPLC-HDMS method (n = 5). Table S2: the detailed information of selected ions from QC samples of the UPLC-HDMS method. Table S3: the detailed information of selected ions from QC samples of the UPLC-HDMS method. [file 2716325.f1.docx]

# HPLC analysis

The concentrations of the alkaloids in the 1500mg/kg/d and 4500mg/kg/d doses of ASAE samples were analyzed by HPLC. The separations were carried out at 25℃ on a cation-exchange column (SCX, particle size 5 μm, 4.6 mm × 250 mm), eluted with the mobile phase consisted of acetonitril-0.2% phosphate solution (1000 mL solution containing with 2 mL phosphoric acid and 2 mL ammonium hydroxide) (72:28). The flow-rate was 1.0 mL/min and the eluent was monitored at 215 nm. In terms of the retention time of the commercial standards, the HPLC chromatogram revealed that there were four alkaloids in the ASAE (shown in Fig. S1), including arecoline (peak 1), guvacoline (peak 2), arecaidine (peak 3), guavacine (peak 4). Their concentrations were showed in Table 1.

1

2

3

4

1

3

4

A

B

2

**Figure S1.** The HPLC chromatograms of mixed standards (A) and 1500 mg/kg/d ASAE samples (B). [1] arecoline; [2] guvacoline; [3] arecaidine; [4] guavacine.

# Validation of the UPLC-HDMS method

The RSD of t_R_ and peak intensities for the selected ions from QC samples were calculated to evaluate the method performance.

**Table S1.** The analytical performance of the UPLC-HDMS method (*n=5*).

| **t_R_-*m/z*** |  | **Precision (RSD,%)** | |  | **Stability (RSD,%)** | |  | **Repeatability (RSD,%)** | |
| --- | --- | --- | --- | --- | --- | --- | --- | --- | --- |
|  |  | **t_R_** | **Peak intensity** |  | **t_R_** | **Peak intensity** |  | **t_R_** | **Peak**  **intensity** |
| **Negative** | | | | | | | | | |
| 1.59-234.20 |  | 0.21 | 5.24 |  | 1.72 | 6.12 |  | 0.67 | 7.22 |
| 2.32-275.38 |  | 0.09 | 6.05 |  | 0.23 | 5.23 |  | 0.13 | 9.31 |
| 7.12-322.57 |  | 0.05 | 4.72 |  | 0.25 | 8.39 |  | 0.25 | 8.14 |
| 16.32-512.33 |  | 0.19 | 4.81 |  | 0.30 | 5.74 |  | 0.02 | 5.20 |
| 17.88-539.14 |  | 0.14 | 7.19 |  | 0.11 | 6.52 |  | 0.32 | 4.92 |
| **Positive** | | | | | | | | | |
| 0.82-253.32 |  | 0.59 | 8.38 |  | 0.81 | 7.07 |  | 0.33 | 7.21 |
| 12.21-376.29 |  | 0.12 | 4.61 |  | 0.02 | 4.19 |  | 0.26 | 4.55 |
| 14.17-478.12 |  | 0.03 | 2.23 |  | 0.14 | 2.11 |  | 0.56 | 4.18 |
| 16.89-502.41 |  | 0.36 | 5.72 |  | 0.44 | 7.45 |  | 0.24 | 2.62 |
| 18.65-632.23 |  | 0.22 | 5.88 |  | 0.82 | 5.46 |  | 0.04 | 3.27 |

**Table S2.** The detailed information of selected ions from QC samples of the UPLC-HDMS method.

| **ESI** | **t_R_** | **Determined** | **Precision** | | **Stability** | | **Repeatability** | |
| --- | --- | --- | --- | --- | --- | --- | --- | --- |
|  | **(min)** | **(m/z)** | **Average intensity** | **SD** | **Average intensity** | **SD** | **Average intensity** | **SD** |
| ESI^+^ | 0.82 | 253.3226 | 218329 | 18296 | 219027 | 15485 | 218959 | 15787 |
|  | 12.21 | 376.2921 | 1178663 | 54336 | 1174921 | 49229 | 1188134 | 54060 |
|  | 14.17 | 478.1205 | 792143 | 17665 | 805288 | 16992 | 809098 | 33820 |
|  | 16.89 | 502.4137 | 86957 | 4974 | 92790 | 6913 | 85991 | 2253 |
|  | 18.65 | 632.2309 | 13635701 | 801779 | 13654612 | 745542 | 13663892 | 446809 |
| ESI^-^ | 1.59 | 234.2011 | 9862911 | 516817 | 9932673 | 607880 | 9935866 | 717370 |
|  | 2.32 | 275.3838 | 5880515 | 355771 | 6027169 | 315221 | 6116908 | 569484 |
|  | 7.12 | 322.5721 | 8173498 | 385789 | 8159231 | 684559 | 8147195 | 663182 |
|  | 16.32 | 512.3307 | 1085603 | 52218 | 1076022 | 61764 | 1064752 | 55367 |
|  | 17.88 | 539.1443 | 10478515 | 753405 | 10390633 | 677469 | 10205170 | 502094 |

# Identification of biomarkers

**Table S3.** The metabolites (47 and 63) and their retention times.

| **ESI** | **Number** | **Determined** | **t_R_**  **(min)** |
| --- | --- | --- | --- |
|  |  | **(m/z)** |  |
| ESI^+^ | 1 | 522.3534 | 12.68 |
|  | 2 | 520.3380 | 11.36 |
|  | 3 | 542.3215 | 11.33 |
|  | 4 | 568.3373 | 11.32 |
|  | 5 | 482.3575 | 18.82 |
|  | 6 | 551.3868 | 20.70 |
|  | 7 | 637.3029 | 21.66 |
|  | 8 | 313.2717 | 21.94 |
|  | 9 | 546.2797 | 17.35 |
|  | 10 | 1073.7476 | 20.66 |
|  | 11 | 532.3342 | 19.37 |
|  | 12 | 438.1946 | 15.64 |
|  | 13 | 416.2133 | 15.64 |
|  | 14 | 120.0801 | 1.55 |
|  | 15 | 464.3113 | 19.24 |
|  | 16 | 216.9217 | 1.32 |
|  | 17 | 524.3309 | 20.41 |
|  | 18 | 483.3613 | 18.82 |
|  | 19 | 513.5049 | 22.56 |
|  | 20 | 638.3062 | 21.66 |
|  | 21 | 466.3265 | 20.94 |
|  | 22 | 1074.7509 | 20.66 |
|  | 23 | 230.2469 | 14.23 |
|  | 24 | 285.2965 | 23.48 |
|  | 25 | 541.5356 | 23.27 |
|  | 26 | 257.2655 | 21.60 |
|  | 27 | 552.3897 | 20.70 |
|  | 28 | 547.2834 | 17.35 |
|  | 29 | 552.3635 | 21.38 |
|  | 30 | 314.2752 | 21.94 |
|  | 31 | 659.2846 | 21.66 |
|  | 32 | 203.0518 | 1.36 |
|  | 33 | 120.0882 | 15.57 |
|  | 34 | 524.2746 | 20.44 |
|  | 35 | 522.3729 | 18.77 |
|  | 36 | 280.0907 | 1.30 |
|  | 37 | 785.5857 | 22.27 |
|  | 38 | 464.2803 | 11.34 |
|  | 39 | 258.2778 | 16.09 |
|  | 40 | 256.8196 | 1.36 |
|  | 41 | 701.5557 | 23.70 |
|  | 42 | 504.3399 | 18.82 |
|  | 43 | 522.2805 | 17.30 |
|  | 44 | 524.269 | 20.44 |
|  | 45 | 218.9186 | 1.30 |
|  | 46 | 520.3801 | 17.50 |
|  | 47 | 483.3256 | 20.34 |
| ESI^-^ | 1 | 255.2332 | 17.34 |
|  | 2 | 279.2322 | 15.30 |
|  | 3 | 303.2324 | 16.28 |
|  | 4 | 253.2168 | 14.43 |
|  | 5 | 283.2635 | 9.14 |
|  | 6 | 494.3231 | 19.42 |
|  | 7 | 409.2359 | 20.00 |
|  | 8 | 167.0205 | 1.35 |
|  | 9 | 407.2801 | 13.62 |
|  | 10 | 480.3084 | 18.38 |
|  | 11 | 506.3224 | 18.82 |
|  | 12 | 508.3387 | 20.54 |
|  | 13 | 270.7972 | 1.33 |
|  | 14 | 552.3655 | 19.32 |
|  | 15 | 266.8039 | 1.33 |
|  | 16 | 1093.7262 | 20.52 |
|  | 17 | 827.5616 | 21.30 |
|  | 18 | 608.3156 | 18.50 |
|  | 19 | 542.3334 | 18.36 |
|  | 20 | 570.3640 | 20.54 |
|  | 21 | 582.3757 | 21.16 |
|  | 22 | 634.3309 | 18.81 |
|  | 23 | 278.8292 | 1.28 |
|  | 24 | 544.3040 | 14.80 |
|  | 25 | 152.8831 | 1.21 |
|  | 26 | 150.8863 | 1.22 |
|  | 27 | 492.3446 | 19.30 |
|  | 28 | 554.3019 | 17.59 |
|  | 29 | 326.7577 | 1.21 |
|  | 30 | 508.3030 | 23.18 |
|  | 31 | 466.3287 | 18.93 |
|  | 32 | 585.3478 | 20.57 |
|  | 33 | 1103.6460 | 18.36 |
|  | 34 | 162.8387 | 1.35 |
|  | 35 | 588.8947 | 1.21 |
|  | 36 | 353.2112 | 19.73 |
|  | 37 | 210.8412 | 1.20 |
|  | 38 | 482.3139 | 18.38 |
|  | 39 | 553.3685 | 19.32 |
|  | 40 | 490.2937 | 20.85 |
|  | 41 | 828.5635 | 21.29 |
|  | 42 | 510.3445 | 20.54 |
|  | 43 | 444.6719 | 1.22 |
|  | 44 | 208.8443 | 1.20 |
|  | 45 | 481.3116 | 20.46 |
|  | 46 | 280.8262 | 1.28 |
|  | 47 | 502.6318 | 1.33 |
|  | 48 | 442.6748 | 1.21 |
|  | 49 | 527.3531 | 18.89 |
|  | 50 | 554.3811 | 21.10 |
|  | 51 | 453.2807 | 18.22 |
|  | 52 | 1104.6489 | 18.40 |
|  | 53 | 152.9957 | 19.07 |
|  | 54 | 524.3349 | 18.89 |
|  | 55 | 583.3791 | 21.16 |
|  | 56 | 1038.6672 | 18.36 |
|  | 57 | 500.6334 | 1.21 |
|  | 58 | 404.1585 | 21.92 |
|  | 59 | 562.5861 | 1.32 |
|  | 60 | 504.6287 | 1.33 |
|  | 61 | 458.2383 | 19.02 |
|  | 62 | 446.6688 | 1.21 |
|  | 63 | 480.2802 | 18.38 |

A potential biomarker with an m/z of 255.2332 is used as an example to illustrate the identification process. First, an accurate mass of the ion was obtained by UPLC LTQ-Orbitrap-MS in ESI^-^ mode (Fig. S2 A). The quasi-molecular ion m/z 255.2332 ([M-H]^-^) were found. Candidates were obtained by searching using the molecular weight 255.2332 Da (negative mode, mass error ≤ 10 ppm) from database such as HMDB (http://www.hmdb.ca/) and METLIN (http://metlin.scripps.edu/). The MSE spectrum of the ion m/z 255.2332 generated ions of m/z 237.0268 and m/z 207.1646 by the continuous loss of H_3_O and CH_2_O (Fig. S2 B). Therefore, the fragmentation mode of palmitic acid was matched more analogously to the observation of a fragment ion in the MSE spectrum of m/z 255.2332.


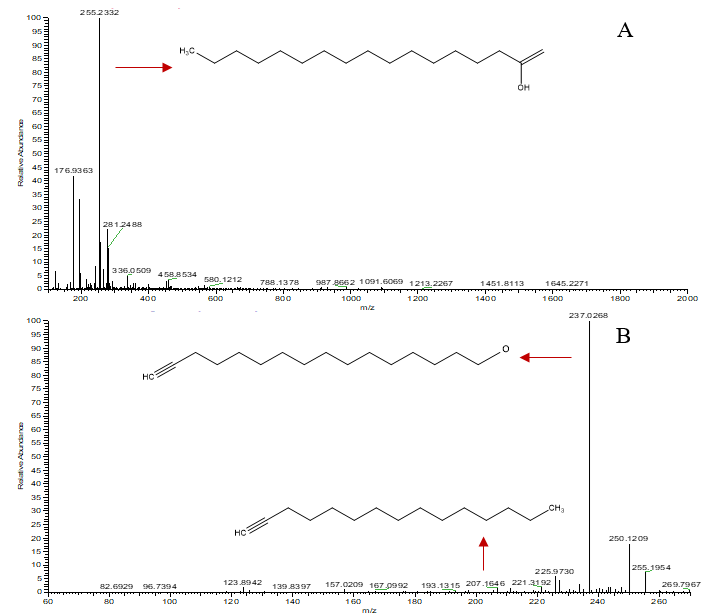


**Figure S2.** LTQ-Orbitrap mass spectra of the metabolite with MSE function at low and high energy for the biomarker at m/z 255.2332 in negative ion mode (A) and the product ion scan spectrum (MSE) for the biomarker in negative ion mode (B).
